# AI-Powered Structural Health Monitoring Using Multi-Type and Multi-Position PZT Networks

**DOI:** 10.3390/s25165148

**Published:** 2025-08-19

**Authors:** Hasti Gharavi, Farshid Taban, Soroush Korivand, Nader Jalili

**Affiliations:** 1 Department of Mechanical Engineering, Southern Methodist University, Dallas, TX 75205, USA; hgharavi@smu.edu; 2 Department of Civil and Environmental Engineering, Southern Methodist University, Dallas, TX 75205, USA; ftaban@smu.edu; 3 Department of Mechanical Engineering, Mississippi State University, Starkville, MS 39762, USA

**Keywords:** structural health monitoring, compressive strength, piezoelectric sensor, machine learning, early-age concrete, non-destructive testing, sensor networks, AI

## Abstract

Concrete compressive strength is a critical property for structural performance and construction scheduling. Traditional non-destructive testing (NDT) methods, such as rebound hammer and ultrasonic pulse velocity, offer limited reliability and resolution, particularly at early ages. This study presents an AI-powered structural health monitoring (SHM) framework that integrates multi-type and multi-position piezoelectric (PZT) sensor networks with machine learning for in situ prediction of concrete compressive strength. Signals were collected from various PZT types positioned on the top, middle, bottom, and surface sides of concrete cubes during curing. A series of machine learning models were trained and evaluated using both the full and selected feature sets. Results showed that combining multiple PZT types and locations significantly improved prediction accuracy, with the best models achieving up to 95% classification accuracy using only the top 200 features. Feature importance and PCA analyses confirmed the added value of sensor heterogeneity. This study demonstrates that multi-sensor AI-enhanced SHM systems can offer a practical, non-destructive solution for real-time strength estimation, enabling earlier and more reliable construction decisions in line with industry standards.

## 1. Introduction

Concrete’s compressive strength is a fundamental property dictating structural capacity and durability [1,2,3]. In practice, this strength is traditionally measured by crushing standard-cured specimens at 28 days (ASTM C39) [3], which is considered the benchmark age for design acceptance. However, waiting 28 days for results can delay critical decisions such as formwork removal or load application [4]. Industry standards acknowledge this issue: for example, formwork supporting structural loads is often kept in place only until the concrete reaches about 70% of its design strength (commonly around 7 days). In fast-track construction, concrete may attain safe load-bearing strength well before 28 days, while in other cases (e.g., cold weather curing or slow-strength-gain mixes), the 28-day strength may still fall short of expectations. This mismatch between standard testing timelines and real strength development highlights a need for reliable early-age strength prediction.

Traditional non-destructive testing (NDT) methods, such as the rebound hammer (ASTM C805) and ultrasonic pulse velocity (ASTM C597), have been used to estimate in situ concrete strength, but each has significant limitations. The rebound hammer provides only a superficial hardness reading that must be empirically correlated to strength; results are influenced by surface condition, moisture, and concrete age [5]. ASTM C805 explicitly cautions that rebound numbers cannot serve as the sole basis for accepting or rejecting concrete due to their variability. Ultrasonic pulse velocity (UPV) techniques can assess material uniformity and detect cracks, but they do not directly measure strength—ASTM C597 states that UPV should not be considered a means of determining compressive strength [6]. Moreover, these conventional NDT approaches typically rely on single-point measurements at a given location and time, providing only a snapshot of the material that may not represent the entire element.

Research gaps remain in leveraging multi-sensor data for early-age strength prediction. Most previous studies and field practices employ one sensor or one type of measurement at a time (e.g., a single embedded “smart aggregate” or a surface test point). This lack of spatial and multi-modal sensing means that valuable information about the heterogeneity and evolution of concrete properties is missed. Curing concrete is not uniform—different sections of a structural element (core vs. surface, center vs. edge) [7,8] can gain strength at different rates. Without multi-position monitoring, early-strength assessment may be unreliable. Additionally, while machine learning has started to improve strength predictions from single sensors or maturity data, few works have combined multiple sensors in different positions with advanced data analytics [9]. There is a clear need to fill this gap by integrating multi-sensor structural health monitoring (SHM) with predictive modeling to capture a more complete picture of strength development.

Addressing these gaps is crucial because improved early-age strength prediction would allow optimization of construction schedules and enhance safety. If concrete strength can be accurately predicted days or weeks in advance of standard tests, project managers can make informed decisions on stripping formwork, tensioning tendons, or applying loads without compromising structural integrity. Early identification of under-strength concrete would trigger timely remedial actions, whereas confident verification of adequate early strength could accelerate construction, reducing downtime. In essence, a reliable predictive system helps ensure that structures are neither opened to service too early (avoiding collapse or damage) nor kept idle longer than necessary (avoiding costly delays).

In this study, we propose a multi-sensor SHM approach using piezoelectric (PZT) sensors and machine learning to predict concrete compressive strength at early ages. Piezoelectric sensors offer the ability to both excite and sense stress waves [10] in concrete; by placing PZT sensors at different positions (and of different types/orientations) on the curing concrete, we capture the concrete’s dynamic impact response signature as it stiffens. When a small impact or vibration is introduced, the resulting wave propagation characteristics (wave speed, resonant frequencies, damping, etc.) are measured by the network of PZTs. These features are directly influenced by the elastic modulus and internal structure of the concrete, which evolve during curing and are related to compressive strength. Prior studies have shown the promise of individual embedded PZT sensors (often termed “smart aggregates”) in monitoring strength gain. Our approach extends this concept to a distributed sensor network, collecting rich, multi-point data that a machine learning model can learn from. By fusing data from multiple PZTs, the model can recognize complex patterns of strength development that single-sensor methods miss. Importantly, this method remains completely non-destructive and in situ, aligning with industry efforts to reduce reliance on extensive cylinder break tests and improve real-time monitoring of concrete (as seen in emerging standards like the maturity method in ASTM C1074, though here we utilize mechanical response data rather than just temperature history).

### Contributions

The core contributions of this study are summarized as follows:

- **Novel Multi-Sensor Monitoring Framework:** Development of a new SHM framework that deploys multiple piezoelectric sensors at different locations on concrete elements to continuously monitor the impact-induced response, providing a more comprehensive view of curing progress than single-point tests.
- **Early-Age Strength Prediction Model:** Implementation of a machine learning model trained on multi-sensor data to predict concrete compressive strength at early ages (well before 28 days), improving the accuracy and lead time of strength estimations compared to traditional NDT or empirical maturity methods.
- **Integration with Industry Standards:** Demonstration of how the proposed system can complement and enhance standard practices (ASTM C39 cylinder tests, ASTM C805 rebound hammer, ASTM C597 UPV, and ACI guidelines for formwork removal) by offering real-time, in situ strength insights. The method is calibrated and validated against standard compressive tests to ensure results are grounded in established benchmarks.
- **Practical Impact on Construction Scheduling and Safety:** Evidence that our multi-sensor approach can reliably determine when concrete reaches key strength thresholds (e.g., 70% of $f_{c}^{\prime }$ for safe formwork stripping), thereby enabling more optimal timing of construction operations and reducing the risk of premature loading or unnecessary delays.
- **Contribution to SHM Research:** Expansion of the SHM knowledge base by introducing multi-position sensing for material property estimation. The study illustrates the benefits of data fusion from multiple sensors in a civil engineering context and provides an open dataset and guidelines for future researchers to build upon this multi-sensor machine learning approach for concrete strength monitoring.

## 2. Methodology

This study aimed to predict the compressive strength of concrete using an NDT method using piezoelectric sensors. Ten concrete cube specimens (15 cm × 15 cm × 15 cm) were prepared using plastic cube molds (Deslauriers, Inc., Roselle, IL, USA) and monitored over a 28-day curing period (Section 2.1). Data were collected on Days 3, 7, 14, 21, and 28 using a rebound hammer (ZC3-A, Antstone, Shenzhen, China) and three types of surface-mounted PZTs arranged in a structured layout (Table 1, Section 2.2). Mechanical impacts were applied to the center of the top surface using the rebound hammer, and the resulting wave responses were captured by a four-channel data acquisition system (Section 2.3). The signals transmitted via BNC to dual hook clip cables (Amadget, Shenzhen, China) were preprocessed (Section 2.4), and key features were extracted to reflect the material’s stiffness-related characteristics (Section 2.5). The actual compressive strength was measured using a standard compression testing machine (Section 3.1), and the extracted features were then used to train supervised machine learning models for strength prediction at various stages of curing (Section 3.2 and Section 3.3).

### 2.1. Materials

Concrete was made using QUIKRETE^®^ 80 lb. Ready-To-Use Concrete Mix (No. 1101), a pre-blended mix of cement, sand, and gravel that provides a compressive strength of at least 4000 PSI (27.4 MPa) and yields approximately 0.6 cubic feet per bag. The dry mix was poured into a concrete mixer and combined with clean water. The consistency of the fresh mix was verified using a slump test in accordance with ASTM C143/C143M-20 [11]. The measured slump was 7.5 cm, which falls within the typical range of 75–100 mm recommended for general-purpose structural concrete, indicating suitable workability for casting and handling.

**Figure 1:**
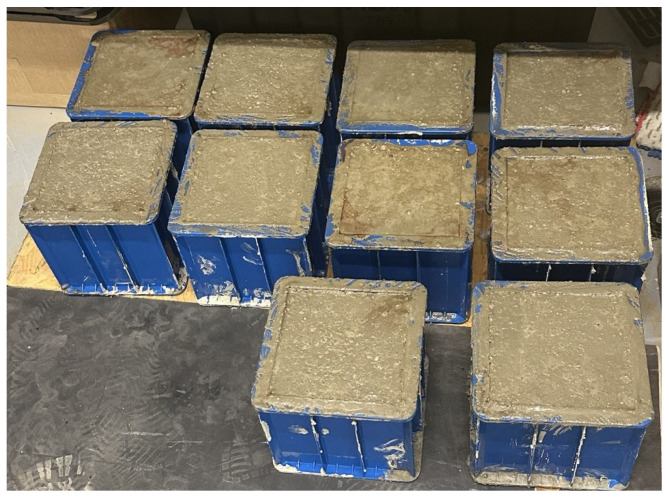
Concrete cube specimens (15 cm × 15 cm × 15 cm) after casting in steel molds. The molds were filled in three layers, each compacted with 35 blows, following ASTM C192.

Each cube mold (15 cm × 15 cm × 15 cm) was filled in three equal layers, with each layer compacted using 35 strokes of a 16 mm steel tamping rod, as recommended in ASTM C192 [12] for preparing concrete specimens, as illustrated in Figure 1, for preparing concrete specimens. The ambient air temperature during casting was around 57 °F (14 °C). After 24 h, the specimens were removed from the molds and placed in a water pond for curing until their scheduled test day. In addition to signal measurements, each concrete sample was broken using a compression testing machine to measure its actual compressive strength. This allowed accurate training and validation of the prediction model. Signals were recorded over a 5 s period, starting at the moment of impact. Each test was repeated three times. On each testing day, two samples were used to ensure reliability. The water and air temperatures were measured before each test, and the weight and size of the samples were recorded. This ensured that the conditions for all samples remained similar. If the compressive strengths of the two samples from the same day were very different, that day’s results were ignored and repeated.

### 2.2. Piezoelectric Sensors and Layout

Each PZT was attached using a thin layer of Loctite quick-set epoxy glue. The glue was applied evenly to avoid air gaps and ensure proper adhesion between the sensor and the concrete surface.

**Figure 2:**
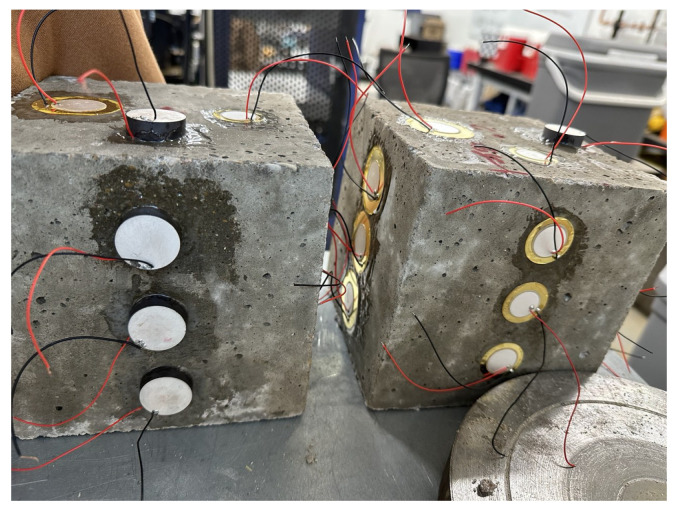
Concrete specimens instrumented with three types of surface-mounted PZTs, as listed in Table 1, using epoxy adhesive. Sensors were attached at different heights (top, middle, bottom) and on the top surface to evaluate signal propagation and sensitivity based on placement and type.

For each sample, one PZT was placed on the top surface, and three others of the same type were attached on the sides at different heights, top, middle, and bottom, as shown in Figure 2. This layout allowed us to evaluate which sensor performed best and how deeply the mechanical wave from the rebound hammer could be detected.

### 2.3. Experimental Procedure

Following standard practice per ASTM C39/C39M [3] and C109/C109M [13] guidelines, compressive strength was tested at 3, 7, and 28 days. We added Day 14 and Day 21 tests to improve temporal resolution and better capture the curing progression. Two cube specimens were tested per day, and multiple PZT-based signal measurements were collected at different positions and repetitions. For each sensor position, we tested and recorded the response three times to ensure consistency and reduce random error. This resulted in 60 total samples, providing sufficient variation for feature extraction and model training.

On each test day, two samples were taken out of the curing pond. The concrete surface was dried and cleaned in preparation for testing. A rebound hammer (Type N, impact energy 2.207 J) was used to apply a mechanical hit to the center of the top surface of each sample. The reason for using the rebound hammer was to ensure that all concrete specimens received the same amount of force under consistent conditions. Since uniform impact strength is important for comparing sensor responses and extracting reliable features, the rebound hammer provided a repeatable and controlled way to deliver identical hits. During each impact, the selected PZTs were connected to the data acquisition device using BNC-to-alligator cables to record the electrical signals generated in response to the mechanical hit.

The DAQ system was a National Instruments NI USB-6229 (NI USB-6229-BNC, National Instruments, Austin, TX, USA) connected to a laptop. The channels AI0 to AI3 were used for signal input. Data acquisition and analysis were performed in Python (version 3.11.7; Python Software Foundation, Wilmington, DE, USA) using the NI-DAQmx Python API (version 0.9.0; National Instruments, Austin, TX, USA), together with NumPy (version 1.26.4), pandas (version 2.1.4), and Matplotlib (version 3.9.1).

Python code was used to control the recording, with a capture time of 5 s.

### 2.4. Preprocessing and Data Preparation

Step 1: Record the signal from all PZTs for each test over a 0 to 5 s time window. This captures the full waveform, including any delay or noise.

Step 2: Remove bias and DC offset from the signal using the full 5 s recording. This step normalizes the baseline and eliminates static distortion.

Step 3: Automatically detect the first and last significant peaks in each sample. This isolates the portion of the signal that contains meaningful wave responses.

Step 4: Crop the signal from the first to the last peak—typically around 0.2 s—to extract a clean segment suitable for feature extraction.

The final data were stored in Excel format and used for later feature extraction and machine learning analysis.

### 2.5. Feature Extraction

To convert raw sensor signals into machine-readable formats, we applied automated time-series feature extraction using the tsfresh Python library (Time Series Feature Extraction based on Scalable Hypothesis tests) [14]. This approach allowed us to compute hundreds of statistical, temporal, and frequency-domain descriptors from the structural health monitoring data collected via the PZT sensors. tsfresh computes a comprehensive set of features such as mean, variance, skewness, energy, entropy, peak counts, autocorrelation, and frequency coefficients in a 5 s time window with a sampling rate of 50 KHz, which capture underlying mechanical wave properties like stiffness changes, damping behavior, and resonant responses during concrete curing. These properties are influenced by the evolving material structure and are known to correlate with compressive strength [15].

This set of extracted features includes not only those commonly used in the literature, such as signal energy, spectral entropy, root mean square, and standard deviation [16], but also extends beyond them by incorporating higher-order statistical moments (e.g., kurtosis, skewness), autocorrelation lags, partial autocorrelations, time-reversal symmetry statistics, and wavelet transform coefficients. These advanced descriptors allow for a more comprehensive characterization of the PZT signal response during curing, capturing subtle variations in material behavior that are not easily detected by traditional metrics. Such a rich and diverse feature set enhances the performance and robustness of the downstream machine learning models in predicting concrete strength development.

## 3. Results

### 3.1. Strength Analysis

**Figure 3:**
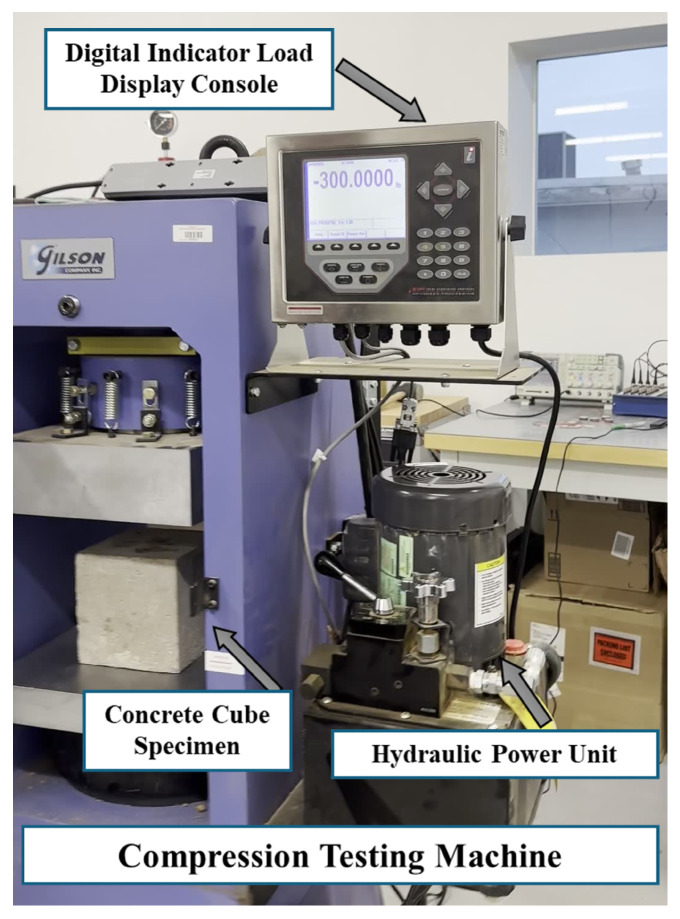
Labeled components of a compression testing machine (Gilson Company, Lewis Center, OH, USA) used for evaluating the compressive strength of concrete specimens. The setup includes a concrete cube specimen placed between loading platens, a Digital Indicator Load Display Console for monitoring applied load, and a Hydraulic Power Unit that drives the loading mechanism.

**Figure 4:**
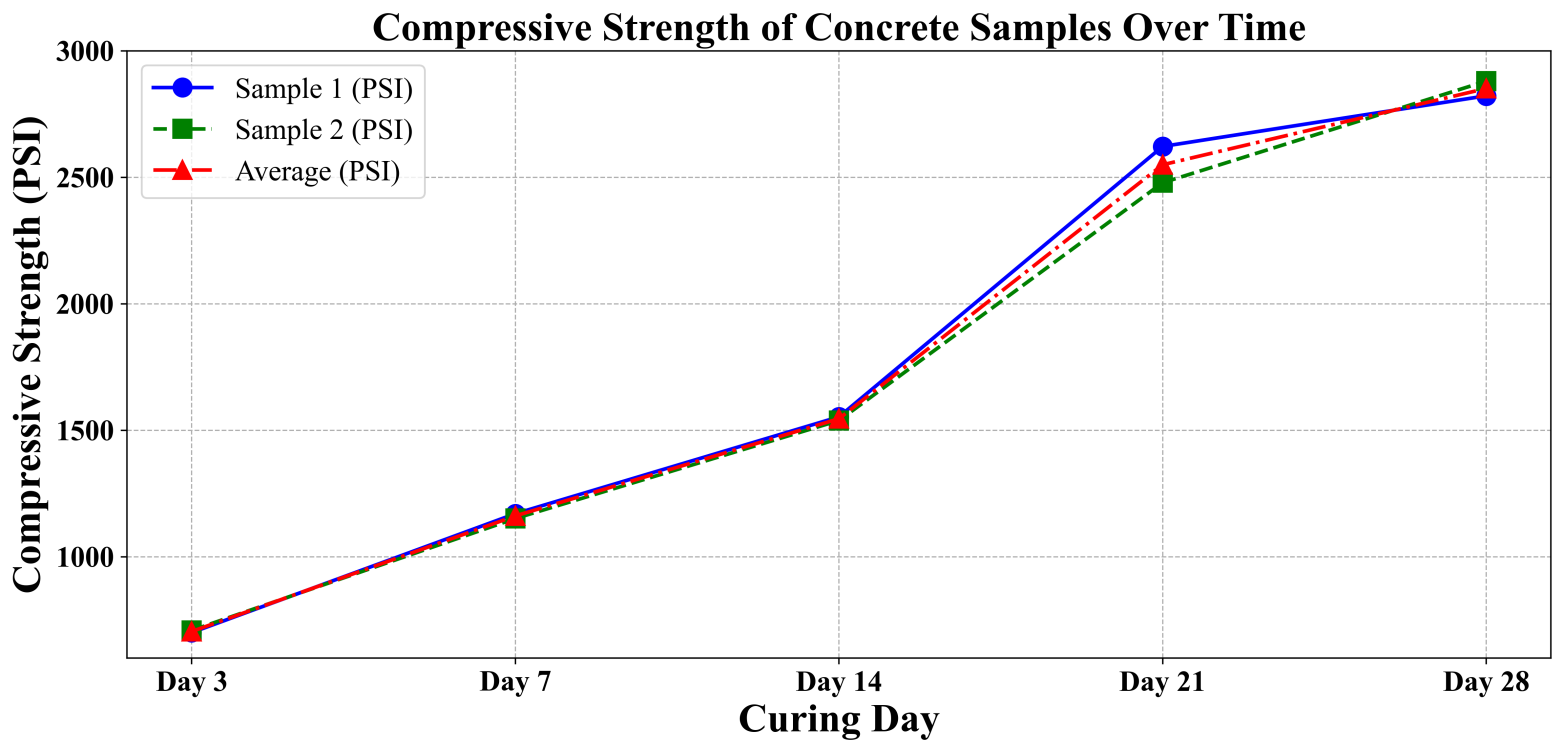
Compressive strength of concrete samples over time.

After data collection, the samples were tested using a compressive testing machine to obtain the actual compressive strength (Figure 3). As shown in Table 2, for each curing day, the difference between the compressive strengths of the two tested samples was assessed to ensure consistency and reliability. According to ASTM C39 [3], if the difference between two test specimens exceeds 8% of their average strength, the results are considered questionable and should be investigated or repeated. In this study, all measured differences remained well within the 8% threshold, confirming the uniformity of specimen preparation and curing conditions. This consistency is further visualized in Figure 4, where the curves for Sample 1, Sample 2, and their average closely follow each other across all curing ages. The strong alignment of the individual sample curves with the average trend confirms minimal variation and validates the integrity of the compression strength data used in the analysis.

### 3.2. Model Performance Using Combined Sensor Data

We evaluated the performance of five machine learning models—Support Vector Machine (SVM), Generalized Linear Model (GLM), Decision Tree, Multi-Layer Perceptron Neural Network (NeuralNet), and Gaussian Naïve Bayes—on two distinct feature sets: (1) the full feature set, consisting of 8755 statistical and frequency-domain descriptors extracted using the tsfresh library, and (2) a reduced subset of the top 200 features selected based on ANOVA F-score rankings. These feature sets were derived from time-series data collected by PZT sensors during the concrete curing process and reflect various signal characteristics linked to material strength evolution.

**Figure 5:**
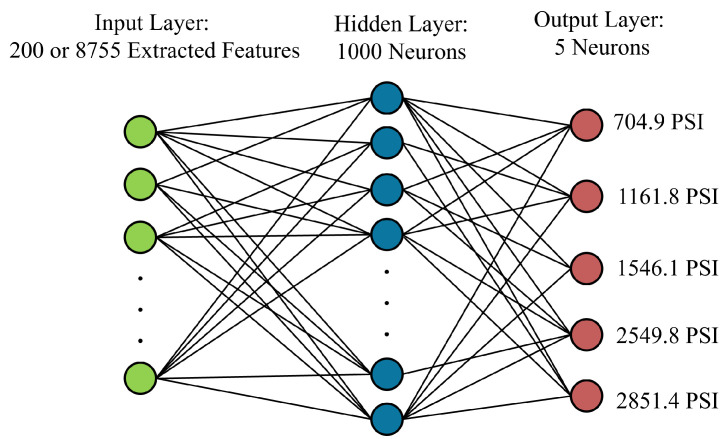
Architecture of the MLP used for compressive strength classification. Input dimensions vary based on the feature set (200 or 8755), with a single hidden layer of 100 ReLU-activated neurons and a five-class softmax output.

Among these models, the Multi-Layer Perceptron (MLP) served as a representative shallow neural network architecture. The MLP was designed to operate on the engineered feature sets and consisted of a single hidden layer with 100 neurons, each using the Rectified Linear Unit (ReLU) activation function to capture nonlinear dependencies within the input data. The output layer comprised five neurons—each corresponding to one of the predefined compressive strength categories—and applied a softmax activation to yield class probabilities. The model was optimized using the Adam optimizer with a maximum of 1000 iterations, enabling robust convergence without overfitting. A schematic overview of the network is shown in Figure 5, illustrating the transformation from input features to output classification.

The classification results reveal that the SVM, GLM, and the NeuralNet all experienced substantial performance improvements when using the top 200 features, each achieving an accuracy of 95%. This demonstrates the effectiveness of focused feature selection in eliminating irrelevant or redundant information, thereby enhancing generalization.

**Figure 6:**
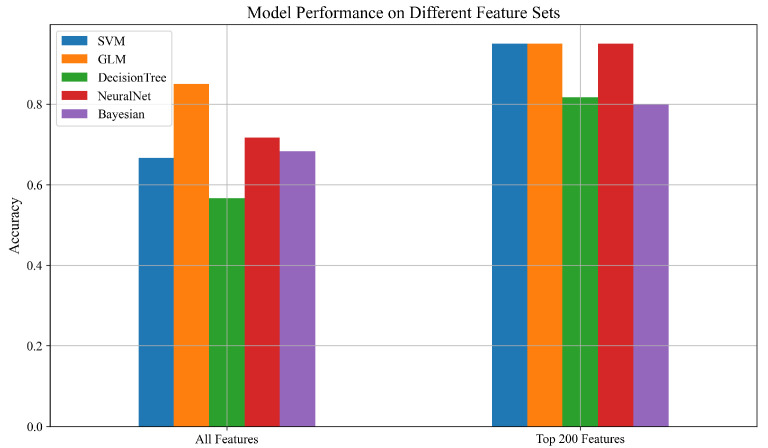
Accuracy comparison of ML models using all features and top 200 ANOVA features.

The Decision Tree classifier also showed a notable improvement, increasing from 78% to 88%, suggesting that it can benefit from targeted feature selection, though potentially to a lesser extent than other models. The Gaussian Naïve Bayes model exhibited a meaningful gain as well, improving from 68% to 80%, indicating that even simpler probabilistic models can benefit from well-curated feature sets. While Table 3 presents the numerical results, the trends, and improvements in model performance with feature selection are more clearly visualized in Figure 6, highlighting the effectiveness of using the top 200 ANOVA-ranked features across different classifiers.

#### 3.2.1. Cross-Validation Accuracy Distribution

**Figure 7:**
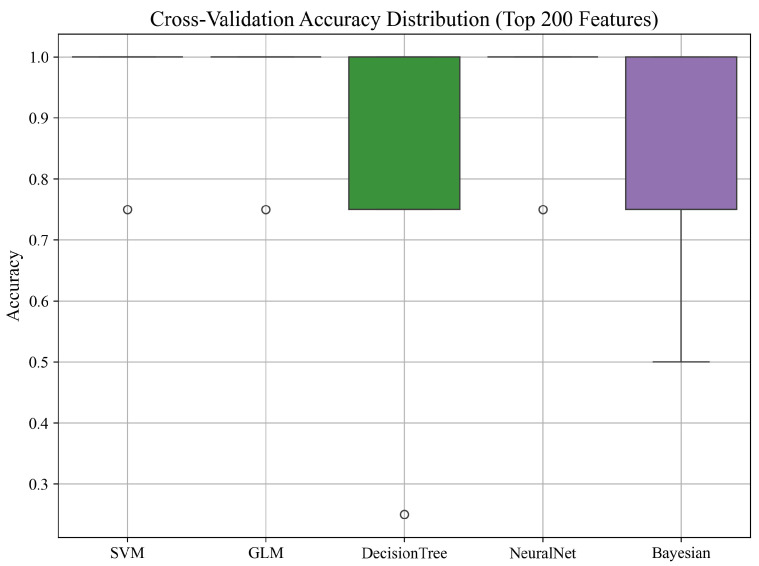
Cross-validation accuracy distribution for all models using the top 200 features from all PZT types.

To evaluate the stability and robustness of the classifiers trained on the top 200 ANOVA-selected features, we performed 5-fold cross-validation using the combined data from all PZT types. The distribution of accuracy scores across folds for each model is illustrated in Figure 7.

As shown in the boxplot, most models exhibited high median accuracy with low variance, indicating consistent performance across folds. The SVM, GLM, and NeuralNet models achieved consistently high accuracy across all splits, with minimal deviation. In contrast, the Decision Tree and Naïve Bayes classifiers showed greater variability, with occasional lower-performing folds. These results further support the reliability of the top-performing models and suggest that careful feature selection contributes to both accuracy and stability in performance.

#### 3.2.2. PCA Visualization of Feature Space

**Figure 8:**
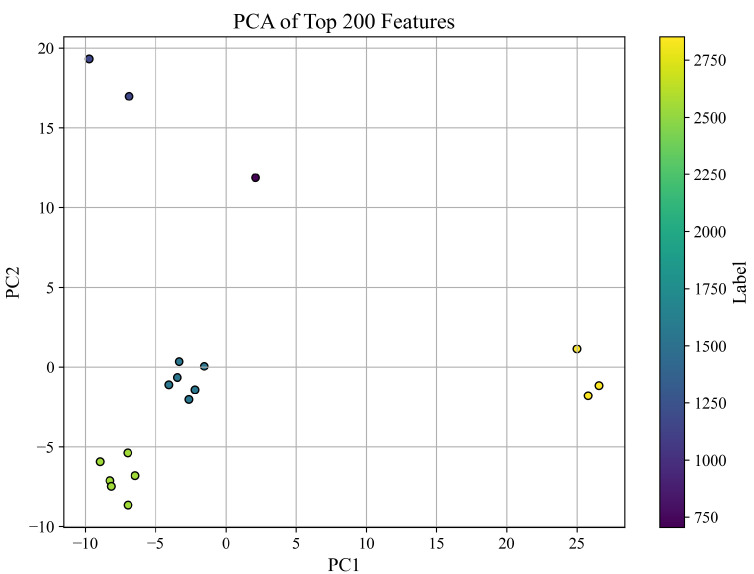
PCA projection of the top 200 features using all PZT types. Colors correspond to compressive strength labels in PSI.

To explore the structure of the feature space, we applied Principal Component Analysis (PCA) to the top 200 ANOVA-selected features extracted from all PZT types. The resulting projection onto the first two principal components is shown in Figure 8, where each point represents a sample and is colored according to its corresponding compressive strength label (in PSI).

The PCA plot provides a two-dimensional representation of the data, which captures the directions of greatest variance. While this projection does not account for all 200 dimensions, it offers a useful qualitative view of how well the top-ranked features separate samples based on compressive strength. Notably, several clusters are visible, and separation patterns emerge that suggest distinguishable trends among different strength ranges.

It is important to clarify that PCA does not rely on only two original features. Instead, it constructs new axes—Principal Components (PC1 and PC2)—as linear combinations of all 200 input features. These components capture the directions of maximum variance in the feature space, with each contributing original feature weighted by its importance to the corresponding component. Thus, the PCA visualization reflects a compressed yet information-rich view of the data, not merely a plot of two selected features.

PCA is a widely accepted technique for visualizing high-dimensional data in two or three dimensions, especially when interpretability of complex patterns is desired. Although this dimensionality reduction does not preserve all feature-level details, it retains the most dominant structural information, enabling qualitative assessment of class separability. For quantitative validation, we rely on classification performance using the full 200 features, as shown in Table 3 and Figure 6.

#### 3.2.3. Feature Importance Analysis

To determine which features contributed most significantly to the prediction of compressive strength, we initially extracted 8755 time-series descriptors from multi-type, multi-position PZT sensor data using the tsfresh library. This comprehensive extraction captured a wide array of characteristics including statistical properties (e.g., mean, variance, entropy), spectral components (e.g., FFT coefficients), and temporal dependencies (e.g., autocorrelations, peaks).

The selection of predictive features was carried out through a structured methodology designed to balance statistical rigor with physical interpretability. First, an initial screening was performed using the ANOVA F-score to identify the top 200 features that exhibited the highest discriminative power with respect to compressive strength. This data-driven ranking provided an objective basis for reducing the feature space from 8755 descriptors.

Subsequently, the selected features were subjected to a qualitative evaluation based on their physical significance in the context of concrete behavior and wave propagation. Features with established relevance—such as entropy, autocorrelation, peak-based metrics, and trend components—were retained for further analysis and model development. Descriptors lacking physical interpretability were not prioritized in the final interpretation, although their statistical contribution was noted for future investigation. This combined approach ensures that the selected features are not only statistically significant but also grounded in meaningful mechanical or signal-based phenomena associated with the material curing process.

**Figure 9:**
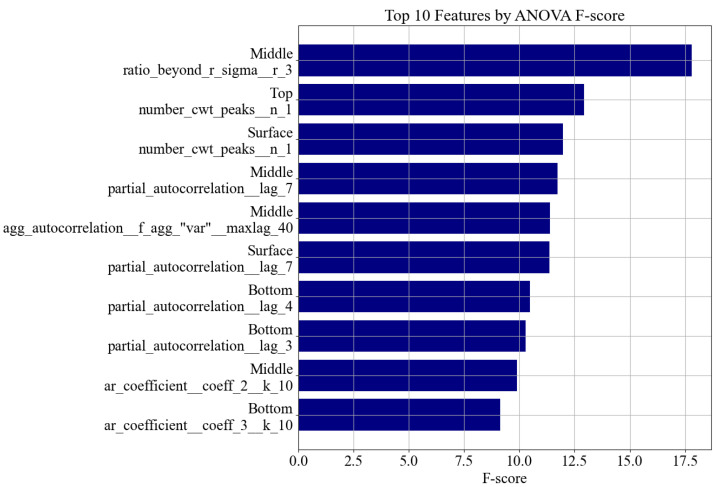
Top 10 most important features selected from all PZT types and sensor positions using ANOVA F-score analysis. Feature names are grouped and wrapped by source position and descriptor.

Figure 9 illustrates the 10 most important features as ranked by ANOVA. These include descriptors such as

- ratio_beyond_r_sigma, which captures signal amplitude dispersion and outliers;
- number_cwt_peaks, indicative of oscillatory behavior;
- partial_autocorrelation and ar_coefficient, which reflect temporal dependency and spectral resonance.

Each of these features carries interpretable physical meaning. For example, an increase in peak count or reduction in entropy typically indicates stiffer material responses during concrete curing. These insights are consistent with prior work in structural health monitoring [17].

Table 4 provides a grouped summary of the top 30 features by PZT position and type, further confirming that discriminative information is spread across the full sensor network. Notably, the surface and middle positions showed particularly strong feature presence, suggesting deeper wave penetration and sensitivity at those locations.

**Figure 10:**
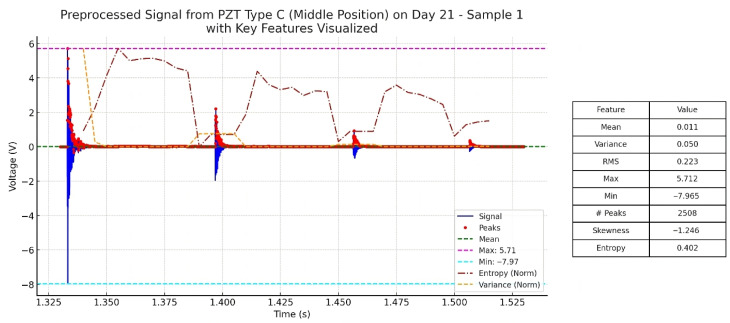
Preprocessed signal from PZT Type C (Middle position, Day 21) with some key features annotated, including normalized entropy, variance, number of peaks, and amplitude extrema.

To enhance interpretability of the extracted features, Figure 10 presents a representative waveform obtained from PZT Type C (Middle position, Day 21), with selected features such as entropy, peak count, and amplitude range annotated. This illustration demonstrates how key statistical descriptors correspond to signal characteristics observed during the curing process, linking quantitative metrics to physical behaviors such as material heterogeneity, signal irregularity, and stiffness development.

**Figure 11:**
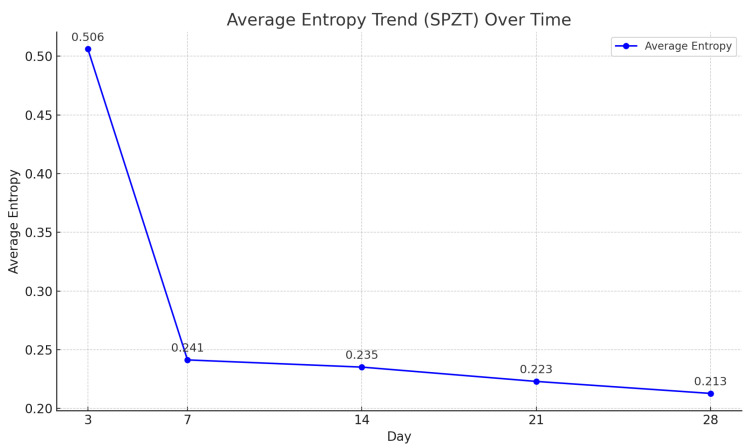
Average entropy trend over time extracted from PZT responses. The decreasing trend illustrates increasing signal regularity and material stiffness during concrete curing.

Further supporting the relevance of selected features, Figure 11 shows how entropy trends over time correlate with material evolution. Entropy decreases as concrete stiffens, while resonance frequency increases, consistent with

(1) $$f_{r}\propto \frac{1}{L}\sqrt{\frac{E}{\rho }},$$

where $f_{r}$ is the resonance frequency, *E* is Young’s modulus, ρ is material density, and *L* is specimen length.

This inverse relationship is also evident through the Shannon entropy formula:

(2) $$H=-\sum_{i=1}^{n}p_{i}\log_{2}p_{i},$$

where $p_{i}$ denotes the normalized spectral power. As stiffness increases, energy becomes concentrated in fewer frequency bins (higher $f_{r}$), leading to lower entropy *H*. Thus, entropy-based and frequency-domain features not only drive accurate prediction but also reflect concrete’s underlying curing dynamics.

#### 3.2.4. Sensor-Wise Contribution of Important Features

**Figure 12:**
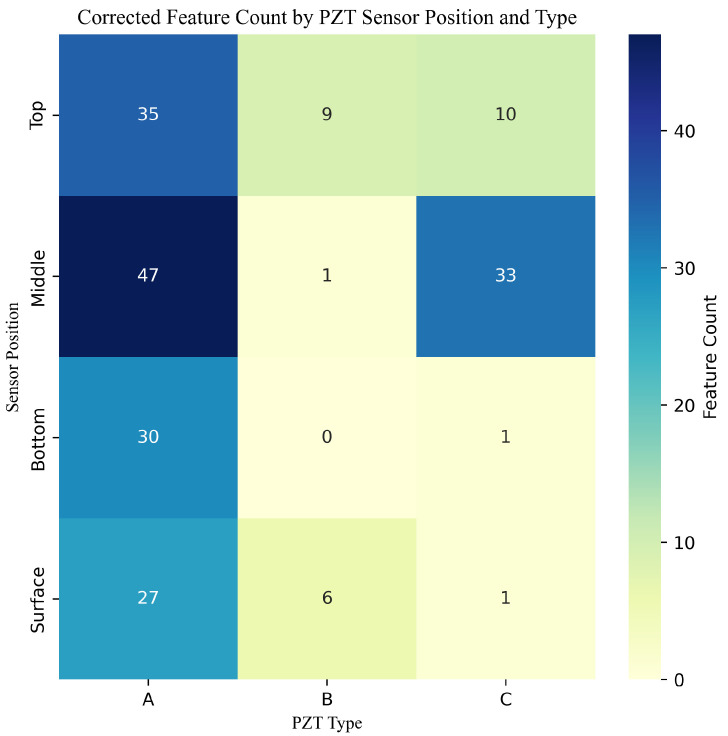
Corrected distribution of the top-ranked features by PZT sensor position and type. Sensor type A contributed the highest number of informative features across all positions, while types B and C showed more selective but complementary contributions.

To further understand the spatial and material sources of predictive information, we analyzed the distribution of the top features with respect to both the sensor location (top, middle, bottom, surface) and the PZT type (A, B, C). A corrected heatmap of feature counts across these categories is presented in Figure 12.

The heatmap reveals that features extracted from PZT type A dominate across all sensor positions, particularly from the middle and top locations. Type C also provided substantial contributions at the middle position, while type B showed more limited but focused relevance—particularly at the top and surface positions. These observations align with the physical and frequency characteristics of the sensors.

PZT A, with a thickness of 8.5 mm and a high resonant frequency of 235 kHz, is better suited to detecting high-frequency components of the stress wave, which are likely more sensitive to subtle changes in stiffness and microstructure during early-stage curing. In contrast, PZT types B and C operate at much lower frequencies (3.5 ± 0.5 kHz and 3–5 kHz, respectively) and have significantly thinner profiles, making them more sensitive to broader, low-frequency wave components. This may explain why PZT C contributed more meaningfully at the middle location—where signal attenuation and internal structural reflections are more prominent—while PZT B’s contributions were more sparse but localized.

These findings support the hypothesis that combining sensors of different frequency sensitivities and placements allows for a richer representation of concrete’s evolving mechanical state. Leveraging this diversity in PZT type and layout enables machine learning models to capture multi-scale information and enhances predictive robustness.

### 3.3. Model Performance by Individual PZT Type

To investigate the individual contribution of each PZT sensor type, we repeated the classification analysis using features derived from only one PZT type at a time (including all four sensor positions associated with that type). This allowed us to assess the predictive power of each sensor independently—on two feature sets:

- All Features: The complete cleaned tsfresh feature set, a total of 2934 features.
- Top 200 Features: A reduced subset selected using ANOVA F-score ranking.

**Figure 13:**
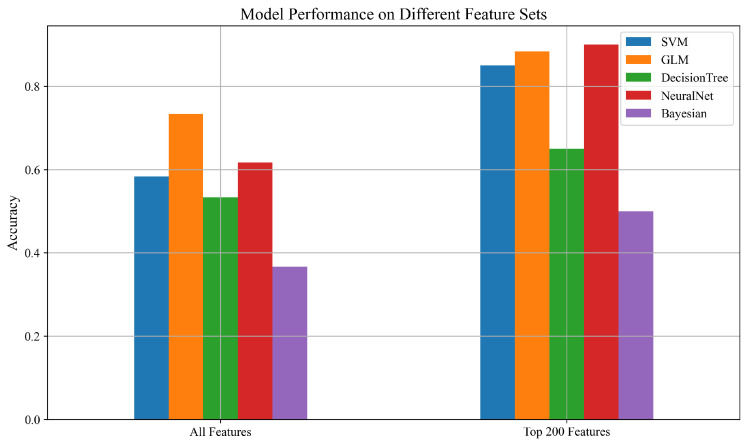
Accuracy comparison of ML models using features from individual PZT types (each including all four sensor positions).

As shown in Figure 13, the classification performance varies across PZT types. In general, the SVM and NeuralNet models showed strong performance when trained on data from certain PZT types, while models like Decision Tree and Bayesian classifiers exhibited more variability. These differences indicate that some PZT types carry more discriminative information about the compressive strength of the concrete samples, possibly due to differences in sensitivity, signal-to-noise ratio, or coupling behavior with the curing concrete.

This analysis highlights the importance of sensor selection in structural health monitoring systems. By identifying the most informative PZT type, future monitoring setups can be optimized for reduced sensor count, improved cost efficiency, and better model performance.

#### 3.3.1. Cross-Validation Performance by Individual PZT Type

To assess the predictive power of each PZT type independently, we conducted a 5-fold cross-validation experiment using features extracted from a single PZT type at a time. For each type, data from all four of its sensor positions (top, middle, bottom, and surface) were included.

**Figure 14:**
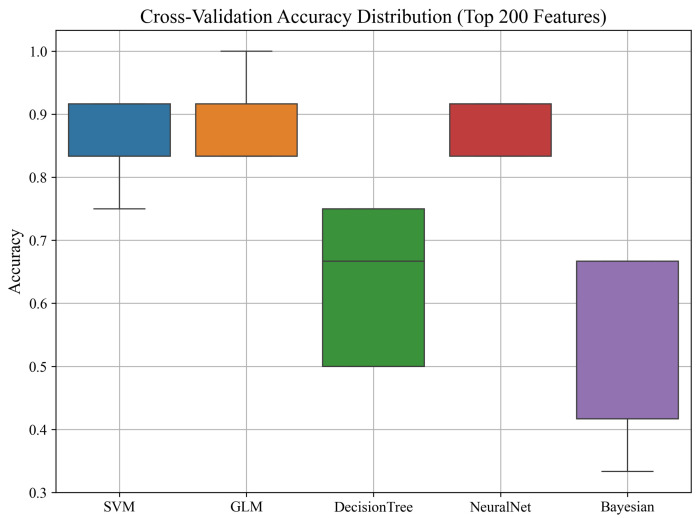
Cross-validation accuracy distribution for each model trained using features from only one PZT type (including all sensor positions).

As shown in Figure 14, the SVM, GLM, and NeuralNet models maintain high and stable performance even when trained using only a single PZT type, indicating that individual PZT types are sufficiently informative for predicting compressive strength. Among the evaluated models, the Decision Tree and Naïve Bayes classifiers exhibited higher variance and lower median performance, suggesting that simpler models may struggle to capture the nuances of the signal when restricted to a single PZT type.

These findings suggest that while high accuracy can be achieved with just one PZT type, especially when using more powerful models, combining data from multiple PZT types (as shown in earlier sections) can further enhance robustness and reduce variability across folds.

#### 3.3.2. PCA Visualization Using a Single PZT Type

**Figure 15:**
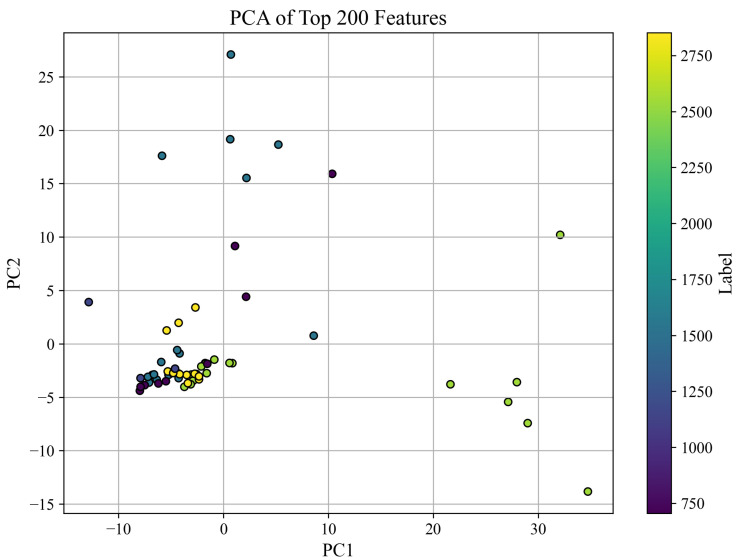
PCA projection of the top 200 features derived from a single PZT type across all sensor positions. Points are colored by compressive strength (PSI).

To assess whether a single PZT type could capture the underlying structure of compressive strength data, we applied PCA to the top 200 features extracted from only one PZT type, incorporating all four sensor positions (top, middle, bottom, and surface). The resulting projection onto the first two principal components is shown in Figure 15.

The PCA visualization indicates that even with features from a single PZT type, samples exhibit a degree of separation based on compressive strength. Clusters corresponding to different strength ranges begin to emerge, though the separation is more diffuse compared to the full PZT combination shown previously. This suggests that while a single PZT type captures meaningful information related to the mechanical state of the sample, incorporating multiple types further enhances feature diversity and the discriminability of the dataset.

#### 3.3.3. Feature Importance Distribution by Sensor Position (Single PZT Type)

To examine the relative contributions of individual sensor positions within a single PZT type, we analyzed the top-ranked features derived exclusively from one PZT type, aggregating across its four sensor positions: top, middle, bottom, and surface.

**Figure 16:**
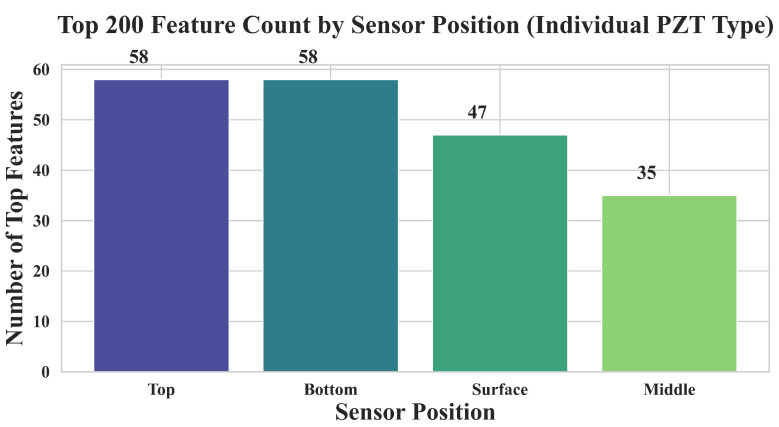
Distribution of the top 200 most important features across sensor positions (top, middle, bottom, surface) from an individual PZT type. The top and bottom positions contributed the highest number of features, followed by surface and middle.

As shown in Figure 16, the top and bottom sensors yielded the highest number of important features, followed by surface and middle. This trend aligns well with the physical layout of the sensor network: top sensors are located nearest to the impact point and thus capture higher-energy, high-frequency responses, while bottom sensors may benefit from deeper wave penetration and reflections from internal structures. The relatively fewer top features from the middle and surface positions suggest that although these sensors contribute useful data, their signal content may be less distinctive in isolating compressive strength.

Moreover, when these features were used independently in the machine learning pipeline, models still performed with surprisingly high accuracy. As seen above, the top 200 features extracted from a single PZT type enabled the SVM, GLM, and NeuralNet classifiers to reach or approach 95% accuracy—comparable to the performance achieved when using all sensor types together. This highlights that even a single PZT type, when monitored across diverse positions, can deliver sufficient signal diversity for robust strength prediction.

These features are shown in Table 5 and represent a variety of signal characteristics—including statistical spread, autocorrelation, peak dynamics, and autoregressive properties—all of which reflect different aspects of the mechanical wave behavior through the concrete. Their position-based origin underscores the value of multi-point sensing, even within a single PZT type.

## 4. Discussion

### 4.1. Interpretation of Model Performance and Feature Selection

The multi-sensor PZT approach improved classification accuracy significantly (90–100%), highlighting the complementary value of different sensor types. Feature selection via ANOVA tests further improved model performance by isolating the most informative features, reducing overfitting, and enhancing interpretability. PCA plots supported these results, showing clear separation between strength classes using selected features.

### 4.2. Methodological Contributions of Automated Feature Engineering

Rather than relying on a limited set of handcrafted features, we used the tsfresh library to automatically extract a broad range of statistical and frequency-domain features from the PZT signals. To reduce dimensionality and improve model performance, we applied a one-way ANOVA test to select the top 200 features with the highest relevance to compressive strength. Among these, we prioritized features with interpretable physical meaning—such as entropy (material disorder), peak structure (wave reflections), and frequency components (stiffness-related resonance). This selection strategy enhanced classification accuracy by filtering out noisy or redundant features while retaining those grounded in concrete behavior and wave propagation. Cross-validation across multiple models confirmed the robustness and generalizability of this automated pipeline.

### 4.3. Implications for Sensor Design and Multi-PZT Arrangements

The strong performance of the multi-PZT system suggests that using a variety of PZT sensors—differing in type or placement—can significantly improve compressive strength prediction. For instance, combining surface and embedded sensors captures both local and bulk responses of concrete, while diversity in resonance frequency can enhance sensitivity to different wave modes. This strategy aligns with findings in related SHM studies that show how sensor networks outperform individual sensors in detecting structural characteristics. Optimizing the number, type, and location of PZTs is therefore crucial for effective monitoring. Although adding multiple sensors increases complexity, our results show that a heterogeneous PZT sensor network enhances model accuracy and can inform more reliable and generalizable strength assessments.

### 4.4. Comparison with Existing Studies and Techniques

This study aligns with recent work demonstrating the effectiveness of PZT sensors and machine learning in predicting concrete strength. Traditional nondestructive tests like UPV and rebound hammer often lack accuracy due to surface sensitivity and limited depth assessment. In contrast, PZT-based methods capture richer signal features more strongly correlated with strength. Sathujoda et al. achieved a prediction accuracy close to 99% using impedance-derived features from embedded PZTs [18]. Jena et al. used a surface-mounted PZT with a deep learning model (2D CNN-BiLSTM), also reaching approximately 99% prediction accuracy, with errors below 2% [16]. Our multi-sensor approach expands on this by fusing data from various PZT types and positions, capturing more diverse signal characteristics. This concept of data fusion is supported by Abazarsa and Yu, who combined ultrasonic and radar NDT to achieve $R^{2}=0.992$, outperforming single methods [19]. Similarly, our use of multiple PZTs improves prediction by leveraging complementary information, reinforcing the value of sensor diversity in concrete monitoring.

### 4.5. Practical Applications in Structural Health Monitoring

This study offers practical insights for field-deployable sensing in civil infrastructure. A PZT sensor network combined with machine learning can serve as an in situ tool to monitor concrete strength in real time, eliminating the need for destructive sampling. For instance, embedding sensors in structural elements (e.g., beams or piers) enables continuous tracking of strength development. Kaur and Negi [20] showed that such embedded sensors can reveal slower strength gain in the core compared to the surface, providing valuable information for decisions like formwork removal. Beyond early-age monitoring, a multi-PZT system could support long-term health monitoring. Over time, the same sensors may detect changes in stiffness or wave propagation caused by micro-cracks, freeze–thaw cycles, or chemical degradation. This enables non-destructive evaluation throughout a structure’s service life. The system could also be deployed in retrofits. Surface-mounted PZTs on existing bridges or garages could provide alerts when signal patterns suggest damage. With proper calibration, the trained model could estimate strength or integrity on-site. While practical use would require durable sensors and possibly edge computing, the computational demand is manageable, making real-time analysis feasible. Overall, this technology can evolve into smart sensing systems, supporting safer, more efficient infrastructure management.

### 4.6. Limitations

Despite promising results, several limitations of this study should be noted:

Sample Size: The dataset was relatively small, which increases the risk of overfitting. The high model accuracy observed may partially reflect the model learning dataset-specific patterns. A larger, more diverse dataset is needed to ensure generalization.

Laboratory Conditions: All tests were conducted under controlled lab conditions (stable temperature, humidity, minimal noise). Real-world environments introduce variables—such as temperature fluctuations, ambient vibrations, and structural complexity—that may affect PZT responses and reduce model accuracy if not accounted for [20].

Sensor Specificity: Only two PZT types were used, each with specific physical and resonant properties. Different sensors or attachment methods could alter signal features, requiring re-calibration. Additionally, long-term durability of sensors (e.g., bonding degradation or signal drift) was not assessed.

Strength Classification vs. Regression: Strength prediction was treated as a classification task based on discrete categories. While practical, this limits the ability to predict precise strength values (in MPa). For continuous estimation or interpolating between classes, a regression-based model may be preferable.

Feature Interpretability: Although ANOVA highlighted important features, many extracted by tsfresh are mathematically complex or abstract. This “black-box” nature may limit interpretability for engineers and pose challenges for field validation or troubleshooting.

### 4.7. Future Research Directions

To broaden the applicability of this study and address current limitations, several directions are proposed:

Dataset Expansion and Field Validation: Future work should involve larger and more diverse datasets, including a significantly increased number of concrete specimens. covering various mix designs, cement types, and curing conditions to improve model generalization. Field deployment in real structures (e.g., bridge elements) under variable environmental conditions is needed to validate system performance beyond lab settings [20].

Sensor Fusion and Network Optimization: Integrating other NDT methods—such as ultrasonic pulse velocity, impact-echo, or laser vibrometry—can complement PZT data and enhance prediction reliability [19]. Research on optimal sensor placement, density, and type (surface vs. embedded) will further improve efficiency.

Real-Time Monitoring and Edge Deployment: Developing real-time systems using microcontrollers or edge computing would enable in situ analysis and early alerts during construction or SHM. These systems could offer on-the-fly predictions and threshold-based warnings for decision support.

Model Transparency and Interpretability: To enhance model interpretability and trust, future work could incorporate explainable AI techniques such as SHAP (SHapley Additive exPlanations) [21] to identify the most influential features and link them to physical phenomena like stiffness or wave propagation [18]. This can help verify that the model’s predictions align with theoretical expectations and support better sensor and feature design. Additionally, the integration of handcrafted features informed by prior studies—such as energy ratios, dominant frequency bands, and wave arrival times—should be explored to complement the automated feature extraction and enhance physical interpretability.

## 5. Conclusions

In conclusion, the study demonstrates a successful integration of multi-sensor data and machine learning for concrete strength prediction. By interpreting the results in light of both data-driven performance and physical reasoning, we provide evidence that a multi-type PZT arrangement, coupled with automated feature extraction and rigorous model tuning, can serve as a powerful nondestructive tool in civil engineering. Addressing the current limitations and pursuing the outlined future directions will move this approach closer to practical field implementation, where it has the potential to significantly enhance how engineers monitor and ensure the quality and safety of concrete structures. The positive comparison to the existing literature and the strong initial accuracy achieved are encouraging signs that with further development, such sensor-informed ML models could become a staple in structural health monitoring and construction quality control [20]. The methodology and insights from this work contribute to the broader vision of smart infrastructure—where real-time data and intelligent algorithms together enable more resilient and informed engineering practices.

## Figures and Tables

**Table 1:** Specifications of the three types of PZT sensors used in the experiment.

Type	Brand/Model (Company, City, Country)	Size (mm)	Resonant Frequency
A	SM111/SMD25T85F234S (STEMINC, Miami, FL, USA)	25 × 8.5	235 kHz
B	DZS Elec (DZS Elec, Shenzhen, China)	27 × 0.15	3.5 ± 0.5 kHz
C	YQBOOM (YQBOOM, Shenzhen, China)	41 × 0.30	3–5 kHz

**Table 2:** Summary of sample compression strengths, differences, and average (PSI).

Day	Sample 1 (PSI)	Sample 2 (PSI)	Difference (PSI)	Average (PSI)
Day 3	700.5	709.2	8.7	704.9
Day 7	1170.5	1151.6	20.3	1161.8
Day 14	1553.4	1538.8	14.5	1546.1
Day 21	2622.2	2478.7	143.6	2549.8
Day 28	2822.4	2880.4	58	2851.4

**Table 3:** Mean classification accuracy (5-fold cross-validation) for different models using all features vs. the top 200 selected features.

Model	All Features	Top 200 Features
SVM	0.67	0.95
GLM	0.85	0.95
DecisionTree	0.78	0.88
NeuralNet	0.76	0.95
Bayesian	0.68	0.80

**Table 4:** Grouped summary of top 30 most important features by PZT location and type.

Sensor Position	*PZT* Type	Example Feature(s)	Category
Top	A	variance_larger_than_std, number_peaks_n_10	Variability, Peaks
Top	C	augmented_dickey_fuller, fft_coeff_57	Stationarity, Frequency
Middle	A	linear_trend_pvalue, skewness, entropy	Trend, Shape, Entropy
Middle	C	spkt_welch_density_coeff_2, fft_coeff_76	Frequency Spectrum
Middle	B	fft_coefficient_real_22	Frequency (Real Part)
Bottom	A	large_standard_deviation, permutation_entropy	Variability, Entropy
Surface	A	duplicate_max, ratio_value_to_length, entropy	Repetition, Complexity
Surface	B	augmented_dickey_fuller_pvalue, ratio_beyond_r_sigma	Stationarity, Spread

**Table 5:** Top 10 most important features from a single PZT type, categorized by sensor position and statistical property.

Feature	Sensor Position	Category
Middle__ratio_beyond_r_sigma__r_3	Middle	Statistical Spread
Top__number_cwt_peaks__n_1	Top	Peak Count
Surface__number_cwt_peaks__n_1	Surface	Peak Count
Middle__partial_autocorrelation__lag_7	Middle	Autocorrelation
Middle__agg_autocorrelation__f_agg_“var”__maxlag_40	Middle	Autocorrelation
Surface__partial_autocorrelation__lag_7	Surface	Autocorrelation
Bottom__partial_autocorrelation__lag_4	Bottom	Autocorrelation
Bottom__partial_autocorrelation__lag_3	Bottom	Autocorrelation
Middle__ar_coefficient__coeff_2__k_10	Middle	AR Coefficient
Bottom__ar_coefficient__coeff_3__k_10	Bottom	AR Coefficient

## Data Availability

The data supporting the findings of this study are not publicly available but are available from the corresponding author upon reasonable request.

## Abbreviations

The following abbreviations are used in this manuscript:

SHM	Structural Health Monitoring
NDT	Non-Destructive Testing
PZT	Lead Zirconate Titanate Piezoelectric, $\mathrm{Pb}\left({\mathrm{Zr}}_{x}{\mathrm{Ti}}_{1-x}\right)O_{3}$
ML	Machine Learning
SVM	Support Vector Machine
GLM	Generalized Linear Model
PCA	Principal Component Analysis
UPV	Ultrasonic Pulse Velocity
ASTM	American Society for Testing and Materials
ACI	American Concrete Institute
DAQ	Data Acquisition
tsfresh	Time Series Feature Extraction based on Scalable Hypothesis tests
